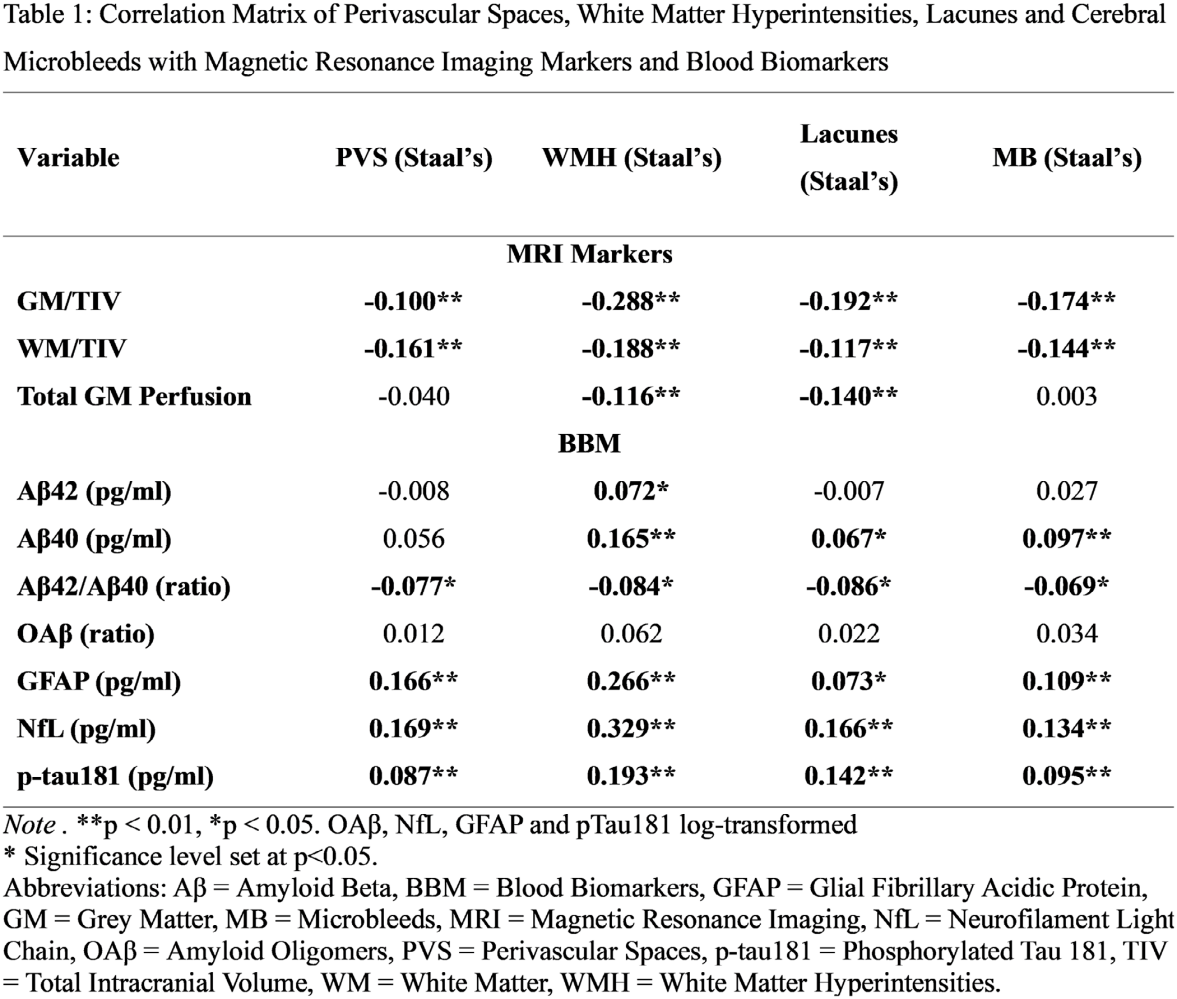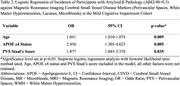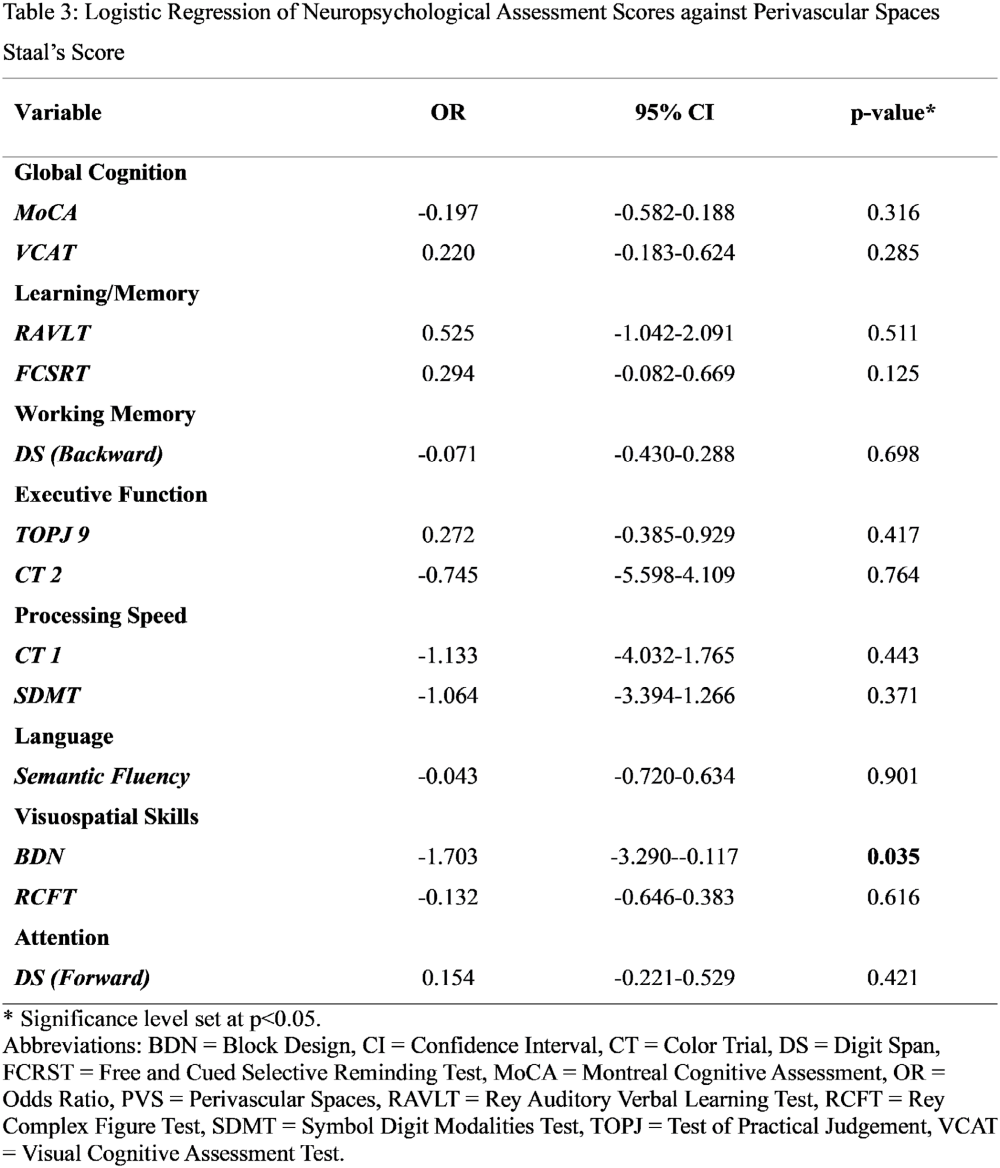# Perivascular Spaces as Early Indicators of Alzheimer's Pathology and Cognitive Impairment in a Southeast Asian Cohort: Insights from the BIOCIS Study

**DOI:** 10.1002/alz70856_097658

**Published:** 2025-12-24

**Authors:** Justin Jit Hong Ong, Yi Jin Leow, Jia Dong James Wang, Nagaendran Kandiah

**Affiliations:** ^1^ Lee Kong Chian School of Medicine, Nanyang Technological University, Singapore, Singapore; ^2^ Dementia Research Centre (Singapore), Lee Kong Chian School of Medicine, Nanyang Technological University, Singapore, Singapore; ^3^ Lee Kong Chian School of Medicine, Nanyang Technological University, Singapore, Singapore, Singapore; ^4^ Neuroscience and Mental Health Programme, Lee Kong Chian School of Medicine, Nanyang Technological University, Singapore, Singapore; ^5^ National Healthcare Group, Singapore, Singapore; ^6^ Duke‐NUS Medical School, National University of Singapore, Singapore, Singapore

## Abstract

**Background:**

Perivascular spaces(PVS), a hallmark of cerebral small vessel disease(CSVD), have emerged as a promising biomarker for vascular contribution to Alzheimer's Disease(AD). While recent research links PVS to cerebrovascular dysfunction, their role in AD pathology and cognitive decline remains understudied, particularly in multi‐ethnic populations. This study evaluates associations between PVS burden in the basal ganglia, AD plasma biomarkers and cognitive impairment in a Southeast Asian cohort, with implications for early dementia detection.

**Method:**

This cross‐sectional study utilized data from the Biomarkers and Cognition Study(BIOCIS), which included 979 community‐based participants in Singapore (mean age:58.2±10.7 years; mean education:14.9±3.5 years; 39.3% male). Comprehensive neuropsychological assessments were conducted, and participants were classified into cognitively normal(CN), subjective cognitive decline(SCD), and mild cognitive impairment(MCI) groups based on established diagnostic criteria. Blood‐based biomarkers of AD, including phosphorylated tau 181(*p*‐tau181), neurofilament light chain(NfL), glial fibrillary acidic protein(GFAP), and amyloid β42/β40 ratio(Aβ42/40) were quantified. Brain imaging was performed using a 3T Siemens Prisma Fit Magnetic Resonance Imaging(MRI) scanner. Markers of CSVD(PVS, white matter hyperintensities(WMH), lacunes, and microbleeds) were graded using validated rating scales. Grading was conducted independently by two raters blinded to cognitive diagnosis, with consensus reached on discrepancies to ensure reliability. Associations between PVS, biomarkers, and cognitive outcomes were analyzed using correlation and multivariable regression models. Analyses were adjusted for age, gender, education years, and apolipoprotein E(APOE) ε4 carrier status.

**Result:**

Higher PVS burden was strongly associated with elevated GFAP(*p* <0.01), NfL(*p* <0.01) and *p*‐tau181(*p* <0.01), and inversely correlated with Aβ42/40 ratio[a lower ratio indicating greater amyloid burden](*p* <0.05). Compared to other CSVD markers, PVS exhibited the strongest association with Amyloid‐β pathology in MCI participants(OR=1.877, *p* = 0.035). Notably, high PVS burden was associated with poorer visuospatial and executive function(Block Design Test, *p* = 0.035).

**Conclusion:**

This study highlights PVS burden as a marker of cerebrovascular dysfunction and amyloid‐tau pathology in preclinical and prodromal AD. As a readily assessable MRI marker, PVS could enhance diagnostic frameworks by complementing cognitive screening, behavioral assessments, and existing CSVD scales. Incorporating PVS into diagnostic protocols may enable earlier and more precise identification of individuals at risk for AD. Longitudinal studies are warranted to confirm its utility as a prognostic biomarker.